# Cognitive—Motor Interference in an Ecologically Valid Street Crossing Scenario

**DOI:** 10.3389/fpsyg.2018.00602

**Published:** 2018-05-03

**Authors:** Christin Janouch, Uwe Drescher, Konstantin Wechsler, Mathias Haeger, Otmar Bock, Claudia Voelcker-Rehage

**Affiliations:** ^1^Faculty of Behavioral and Social Sciences, Institute of Human Movement Science and Health, Chemnitz University of Technology, Chemnitz, Germany; ^2^Institute of Physiology and Anatomy, German Sport University Cologne, Cologne, Germany

**Keywords:** multitasking, dual-tasking, aging, walking, cognitive-motor interference, ecological validity, virtual reality, street crossing

## Abstract

Laboratory-based research revealed that gait involves higher cognitive processes, leading to performance impairments when executed with a concurrent loading task. Deficits are especially pronounced in older adults. Theoretical approaches like the multiple resource model highlight the role of task similarity and associated attention distribution problems. It has been shown that in cases where these distribution problems are perceived relevant to participant's risk of falls, older adults prioritize gait and posture over the concurrent loading task. Here we investigate whether findings on task similarity and task prioritization can be transferred to an ecologically valid scenario. Sixty-three younger adults (20–30 years of age) and 61 older adults (65–75 years of age) participated in a virtual street crossing simulation. The participants' task was to identify suitable gaps that would allow them to cross a simulated two way street safely. Therefore, participants walked on a manual treadmill that transferred their forward motion to forward displacements in a virtual city. The task was presented as a single task (crossing only) and as a multitask. In the multitask condition participants were asked, among others, to type in three digit numbers that were presented either visually or auditorily. We found that for both age groups, street crossing as well as typing performance suffered under multitasking conditions. Impairments were especially pronounced for older adults (e.g., longer crossing initiation phase, more missed opportunities). However, younger and older adults did not differ in the speed and success rate of crossing. Further, deficits were stronger in the visual compared to the auditory task modality for most parameters. Our findings conform to earlier studies that found an age-related decline in multitasking performance in less realistic scenarios. However, task similarity effects were inconsistent and question the validity of the multiple resource model within ecologically valid scenarios.

## Introduction

Many daily activities require us to manage sensory-motor tasks while we simultaneously engage in cognitive tasks. One prominent example is pedestrian mobility, such as walking down a sidewalk while avoiding a collision with another pedestrian, walking while screening items in a shop window, or crossing a non-signalized street while paying attention to relevant traffic information. These activities become even more complex with the advent of portable technologies such as smartphones or music players. In June 2013 the Pedestrian Survey Infographic published, that from over 1,000 American respondents three out of five (60%) 18–65 year olds stated to use smartphones while crossing a street, even though this was considered as dangerous by 70% of these respondents (Liberty Mutual Insurance, 2013).

Standardized laboratory paradigms have provided evidence that sensory-motor performance decreases under dual-task conditions, and that this decrease is especially pronounced in older adults (Kray and Lindenberger, 2000; Verhaeghen et al., 2003). This has been often explained by sensory-motor and cognitive declines within the aging process (Baltes and Lindenberger, 1997; Li and Lindenberger, 2002). Performance decrements in dual-task situations have even been observed with tasks that are considered to be highly automated, like gait. It has therefore been argued that even gait requires cognitive control and higher-level resources (Hausdorff et al., 2005; Yogev-Seligmann et al., 2008). Especially in older adults, gait seems to place high attentional demands and requires more cognitive resources (Lindenberger et al., 2000; Woollacott and Shumway-Cook, 2002; Hausdorff et al., 2008) which leads to greater dual-task decrements in this age group (Al-Yahya et al., 2011).

Performance decrements in dual-task situations have been interpreted in light of several theoretical positions, such as capacity models of attention (Kahneman, 1973) or multiple resource models (Wickens, 2002). In both types of models, two or more tasks compete for common resources, either within a limited attentional resource pool (Kahneman, 1973) or within multiple resource pools (Wickens, 2002). In the latter case, pools are thought to be devoted to different stimulus modalities, signal codes, processing stages, and response channels (Wickens and McCarley, 2007). Both theoretical approaches share the idea that performance deteriorates when the competing tasks are so complex that their combined resource demand exceeds the available resource capacity. The multiple-resource model additionally posits that the tasks must be similar enough in order to compete for the same resource. The determinants of dual-task decrements therefore are task complexity and—in case of the multiple-resource model—task similarity.

Several studies provided evidence for the role of task similarities. They documented interference between tasks that share sensory modalities, processing levels or information channels (Allport et al., 1972; Isreal et al., 1980; Duncan et al., 1997; Talsma et al., 2006). In a street crossing context, such interference could emerge when two tasks require to simultaneously process similar visual signals. This is the case, e.g., when we look for a suitable gap in traffic and concurrently read walking directions on a mobile phone. In contrast, looking for gaps while listening to walking directions over headphones should cause less interference.

So far, most available knowledge about dual-task performance came from traditional laboratory-based research that offers a high controllability and standardization, but lacks ecological validity. Even if real walking is required, tasks are often executed within a laboratory surrounding and most of the applied loading tasks are rather abstract like verbal fluency or arithmetic subtraction tasks. For example, participants were asked to memorize word lists while walking (walk as accurately and quickly as possible on two narrow tracks with different path complexity/avoid obstacles) (Lindenberger et al., 2000; Li et al., 2001). The results revealed diminished performance when the tasks were performed concurrently. Age-related differences were more pronounced in the memory task than in the walking task. This result was discussed as older adults prioritizing walking over memorizing to protect themselves from falls, a view known as “posture first hypothesis” (Shumway-Cook and Woollacott, 2000; Schaefer and Schumacher, 2011; cf. Li et al., 2012 for discussion of mixed results).

Everyday life typically differs from traditional laboratory paradigms in that behavior is uninstructed and volitional, with varying and often unpredictable stimuli and with a wider range of possible and purposeful responses. Little is known about the transferability of laboratory outcomes to more realistic settings. Available literature documents marked differences between laboratory and realistic behavior with respect to gait (Bock and Beurskens (2010), manual grasping (Bock and Züll, 2013) and cognitive performance (Verhaeghen et al., 1993). In a systematic review on dual-task training effects in older adults, Wollesen and Voelcker-Rehage (2014) found heterogeneous results regarding the transferability of training effects to everyday situations. Consequently, several authors cautioned against generalizing laboratory results to real life (Chaytor and Schmitter-Edgecombe, 2003; Li et al., 2005) and questioned the extent to which especially age-related decays apply on everyday-like behavior (Verhaeghen et al., 2012).

Given the above considerations, it seems desirable to expand dual-task research by using more ecologically valid paradigms, without giving up the advantages of a laboratory setting such as controllability and standardization. A promising approach to do so can be seen in virtual reality (VR) settings (Lopez Maite et al., 2016) which can provide an everyday-like, controllable and safe surrounding that can be adapted to the need of the experimenter. Thus, a realistic walking task (e.g., walking down or crossing a street) can be combined with a realistic loading task (e.g., watching for vehicles or using a smartphone) while ambient stimuli are controlled for and relevant measures are extracted. Indeed, several VR pedestrian street crossing studies have been conducted recently (Dommes et al., 2014; Schwebel et al., 2014; Morrongiello and Corbett, 2015). However, most of these studies did not address multitasking (Dommes et al., 2014; Schwebel et al., 2014; Morrongiello and Corbett, 2015) and those which did rather focused on children and young adults than older adults (Chaddock et al., 2011, 2012; Byington and Schwebel, 2013; Gaspar et al., 2014; Tapiro et al., 2016) or used only a single loading task throughout the whole experimental block or session (Neider et al., 2011). For example, Neider et al. (2011) confirmed that dual-task crossing performance deteriorates in old age, but did not evaluate performance changes of the cognitive loading task (cell phone conversation) to control for interaction effects or possible prioritization strategies.

The present study aims to overcome the mentioned limitations by combining a VR street crossing task with a realistic loading task. We posit that the ecological validity of our approach exceeds that of earlier approaches. Specifically, loading tasks are administered either through the visual or the auditory modality, in order to scrutinize the validity of the multiple resource model (Wickens, 2002) for ecologically valid settings. We hypothesized that street crossing requires visual resources and therefore will interfere with visually presented loading tasks more than with auditorily presented loading tasks, particularly in older persons. In accordance with the posture first hypothesis (Lindenberger et al., 2000; Li et al., 2001; Schaefer and Schumacher, 2011), we expected that age-related deficits will be less pronounced for walking than for loading task performance, even in an ecologically valid setting.

## Methods

### Participants

The study was conducted within the DFG (German Research Foundation) Priority Program SPP 1772 “Multitasking,” In total, 134 healthy men and women between 20 and 30 (*n* = 69) and 65 and 75 (*n* = 65) years of age who actively participated in traffic as drivers as well as pedestrians were recruited. Younger participants were recruited via mailing lists from the student pool of the Chemnitz University of Technology (Germany) and the German Sport University Cologne. Older adults were acquired via local newspaper advertising and (only in Chemnitz) further via the participant pool of the Cognition, Brain, and Movement Lab of Chemnitz University of Technology. About half of the young and old participants were recruited and tested in Chemnitz and the other half in Cologne. Both locations used standardized and indentical set ups, test designs and instructions as well as identical hardware and software.

Interested persons were screened in an initial telephone interview for the following exclusion criteria: (a) age range violations, (b) former or current health impairments (heart attacks, brain injuries, strokes; motor impairments that inhibit the participant to continuously walk for 30 min, eye diseases or current relevant injuries), (c) obesity (Body Mass Index, BMI > 30), and (d) driving irregularity (driving a car less than once a week). Further exclusion tests were performed on participant's first laboratory test session (cf. below). No person had to be excluded based on these tests. Before testing began, participants obtained medical clearance from their local physician and signed an informed consent statement to our study. This experiment was part of a larger project in which the same participants were additionally given a car-driving test (reported in another contribution to this issue) and a cardiovascular fitness test. The project was approved by the Ethics Committee of the German Sport University, Cologne.

Six participants dropped out over the study time without giving reasons, three had to be excluded because of simulator sickness, and one participant left the study for personal reasons. The remaining 124 participants were subdivided with respect to age into 61 older adults (OA) with a mean age of 69.97 (*SD* = 2.96) years [females: *n* = 22; BMI = 25.09 (*SD* = 2.44); MMSE = 29.15 (*SD* = 0.85)] and 63 young adults (YA) with a mean age of 23.17 (*SD* = 2.83) years [females: *n* = 40; BMI = 22.04 (*SD* = 2.30); MMSE = 29.67 (*SD* = 0.62)]. OA received 15 € per session as monetary compensation (60 € in total) and YA received course credits. Further, all participants received an individual report of their cardiovascular fitness test as compensation.

### Laboratory screening

Normal hearing was assessed by the Freiburg speech intelligibility test (Freiburger Sprachverständlichkeitstest) with a set cutoff word recognition rate of 50 %. Normal vision was assessed by the Freiburg Visual Acuity Test (FrACT; version 3.9.0) with a cutoff score of 20/60 since driving is presumed to be safe above that score (Keeffe et al., 2002). Lack of visual-field deficits was confirmed by the online version of the Damato Multifixation Campimeter (Damato and Groenewald, 2003). All participants who used visual and hearing aids in their daily life did so in testing as well. Normal overall cognition was assessed by the Mini-Mental State Examination (Folstein et al., 1975) with a cutoff score of 27/30. Finally, the Edinburgh Handedness Inventory (Oldfield, 1971) was used to determine hand dominance. Five participants were left handed, one was ambidextrous but preferred the right hand for typing, and all others were right handed.

### Apparatus and setup

Hardware for the street crossing task consisted of a non-motorized treadmill (DRAX, Speedfit 1000c, Vibrafit®, Solms) and three 46″ TV flat screens that featured a 195 degree horizontal field of view. Treadmill speed was registered opto-electronically, and was synchronized with a first person perspective view of a 3D world. Thus, as participants walked at their own pace, sped up, and slowed down, their viewpoint in the visual 3D world moved accordingly. To reduce physical exertion, participants were asked not to run. For safety reasons, each participant was equipped with a drop guard and asked to keep the non-dominant hand on the treadmill's handrail for the entire test duration.

Headphones (Shark Zone H10 Gaming Headset, Sharkoon Technologies GmbH, Linden, Germany) were used to deliver auditory stimuli and a microphone to register verbal responses. A keypad with 2 × 3 digits was attached within easy reach of the participants' dominant/preferred hand to register manual typing responses.

Software consisted of a modified, commercially available driving simulator (Carnetsoft®, version 8.0 Groningen, NL) that was adapted to the needs of a street crossing task: it displayed the 3D model of a city street from a first person perspective (see section Street Crossing Task).

**Figure 1:**
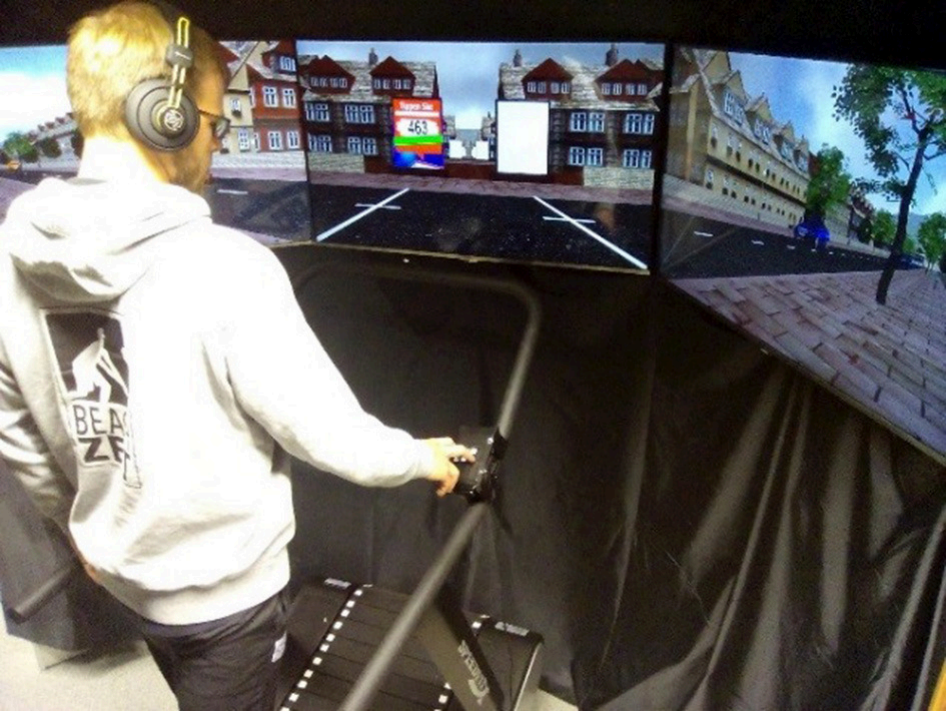
Street crossing simulator set up.

Figure 1 illustrates the set up and displays the modeled city street.

### Street crossing task

The street crossing task was designed similarly to a study by Neider et al. (2010), in which the participant's task was to safely cross a street presented in virtual reality. To do so, they had to detect suitable gaps between the oncoming vehicles. In our scenario, the street consisted of one three-meter wide lane in each direction and was flanked by typical downtown buildings. Vehicles traveled along both lanes at 50 km/h, which is the legal driving speed in German towns. At the onset of each trial, participants walked 15 m through a virtual back alley to reach the curb of the street, stopped, watched for a suitable intervehicle gap and then crossed. Intervehicle gaps increased during each trial according to the sequence 2, 2.5, 3, 3.5, 4, 4.5, 5, 5.5, 6 s, and this sequence was repeated if participants did not cross the street yet. Cars on the far lane reached the crossing area one second later than those on the near lane. Pilot work yielded that this traffic pattern allows safe crossing even for older participants. Far lane traffic was implemented to detect possible behavioral anomalies during the crossing process (e.g., stopping in the middle of the street to let pass the traffic on the far lane before continuing crossing). However, as no such anomalies occurred and analyses of near and far lane provided equally results, only near lane analyses will be reported. A crossing trial was completed when participants reached the opposite walkway, when they caused an accident or when 80 s elapsed.

### Loading task

We used two realistic loading tasks that resembled rehearsal of a shopping list (shopping task) and smartphone usage (typing task). Each given loading task was presented repeatedly from trial onset until trial end to ensure that the crossing task and the loading task could not be dealt with sequentially. Loading tasks were presented visually on some, and auditorily on other trials.

In the *shopping task*, grocery products were sequentially presented either visually on billboards across the street or auditorily through headphones. In the *typing task*, three-digit numbers were sequentially presented either on the billboards (for 4 s each) or through headphones, for about 1.7 s each. Participants reacted by depressing, with their preferred hand, the corresponding numbers on a keypad that was attached to the treadmill handrail. Task type (shopping, typing) and task modality (visually or auditorily) varied quasi-randomly between trials, with the constraint that each type^*^modality combination was presented a total of ten times. To limit the complexity of the present paper, we decided to focus our analyses on the typing task. However, it is important to note that this task was not administered alone but rather intermixed with the shopping task, to mimic the diversity of everyday multitasking. Possible switching costs, resulting from such a loading task intermix will be discussed in a car driving simulator study by Wechsler et al. (under review).

The simulation offered three task conditions. In the control condition “single-task crossing (STcross),” participants walked on the treadmill and crossed the virtual street without loading tasks. In the condition “single-task loading,” participants stood still on the treadmill while the virtual reality display advanced automatically and the loading tasks were displayed sequentially. In this condition, each type^*^modality combination [typing auditory (STtype_aud), typing visual (STtype_vis), shopping auditory (STshop_aud) and shopping visual (STshop_vis)] was presented with a total of 10 trials. In the condition “multitask,” participants walked on the treadmill and crossed the virtual street while concurrently engaged in a loading task (MTtype). Again, there where 10 trials of each type^*^modality combination [auditory typing task (MTtype_aud), visual typing task (MTtype_vis), auditory shopping task (MTshop_aud), and visual shopping task (MTshop_vis)].

Ten control trials of STCross were randomly intermixed with ten trials each of MTtype_aud, MTtype_vis, MTshop_aud, and MTshop_vis. The total of 50 trials was presented in blocks of ten trials each that were characterized as active blocks in which participants had to actually walk on the treadmill. These active blocks alternated with passive blocks of ten single-task loading trials each in which the participants stood still on the treadmill. The latter blocks were formed by intermixing ten trial each of STtype_aud, STtype_vis, STshop_aud, and STshop_vis. The alternation of active and passive blocks was introduced to avoid fatigue. All participants received the same sequence of trials, which took about 40 min.

### Procedure

All data were collected in four sessions of about 2 h each, scheduled one to seven days apart. The first session included a screening (see above) and a familiarization phase in which participants walked on the treadmill. This phase ended when participants and experimenter considered starting, walking and stopping on the treadmill to be smooth and effortless, which took about 10–15 min for YA, and 15–20 min for OA. Afterwards, participants received one practice trial each for STtype_vis, STtype_aud, and STcross. MTtype was not practiced.

Street crossing performance was registered in one of the remaining three test sessions, depending on participant's test order randomization. It was assured that the street crossing experiment was never scheduled on the same day as the cardiovascular fitness test, to avoid fatigue.

### Data reduction

The following performance measures were calculated.

*Back-alley Speed (km/h):* Mean velocity of walking toward the curb. Triggers were set at trial onset, and when participants stopped at the curb.

*Stay Time (s):* Length of time that participants stood still at the curb while watching traffic. Time triggers were set when treadmill pace dropped to 0 m/s and when treadmill pace exceeded 0 m/s thereafter.

*Crossing Speed (km/h)*: Mean velocity of crossing the street. Triggers were set when participants left the curb to cross (treadmill pace > 0 m/s) and when they reached the opposite curb.

*Crossing Failures:* Percentage of unsuccessful trials, as a result of timeouts (i.e., participant did not complete street crossing within 80 s) or experienced a collision (i.e., participant was hit by a car).

*Gap Number:* Serial order of the gap selected for crossing.

*Typing Accuracy (%):* Percentage of trials on which all three digits were typed correctly.

*Typing Reaction Time* (*ms*): Interval from stimulus onset until typing the first digit.

*Multitasking Effects, MTE:* Relative performance change under multitask conditions, with negative values indicating poorer performance (cf. Kelly et al., 2010; Plummer and Eskes, 2015). For *Back-alley Speed, Crossing Speed*, and *Typing Accuracy* MTE was calculated as

(1) $$\begin{array}{r} MTE=\frac{\text{Multitask performance}-\text{Single task performance}}{\text{Single task performacne}}\;x\;100\% \end{array}$$

while for Stay Time, Crossing Failure, Gap Number, and Typing Reaction Time it was calculated as

(2) $$\begin{array}{r} MTE=\;-\;\left(\frac{\text{Multitask performance}-\text{Single task performance}}{\text{Single task performancne}}\;x\;100\%\right) \end{array}$$

### Statistical analyses

Outliers were eliminated by applying the ±3.29 *SD* criterion (Tabachnick and Fidell, 2001), separately for each participant and task. Data were then averaged across repetitions if at least seven repetitions remained, which was the case for all 124 participants.

Each street crossing parameter was submitted to an analysis of variance (ANOVA) with Age (OA, YA) as between-subject factor and Condition (STcross, MTtype_aud, MTtype_vis) as within-subject factor. Typing parameters were submitted to an ANOVA with Age (OA, YA) as between-subject factor and Condition (STtype, MTtype) and Task Modality (visual, auditory) as within-subject factors.

Each MTE score was tested against zero with one-sample *t*-tests in case of normal distributions, and Wilcoxon Signed Rank tests otherwise. Further, MTE scores were submitted to an ANOVA with Age (OA,YA) as between-subject factor and Parameter Type (Stay Time, Walking Speed, Crossing Speed, Failures, Gap, Typing Accuracy and Typing Reaction Time) as well as Task Modality (visual, auditory) as within-subject factors.

Effect sizes are reported as partial eta squares. Homogeneity of variances was determined by Mauchly-tests and, if the sphericity assumption was violated, Greenhouse-Geisser adjustments were applied. When omnibus ANOVA was significant, Bonferroni *post-hoc* tests were conducted. All statistical analyses were conducted with SPSS for Windows, version 25 (IBM Corp., Armonk, NY, USA).

## Results

### Crossing task

**Figure 2:**
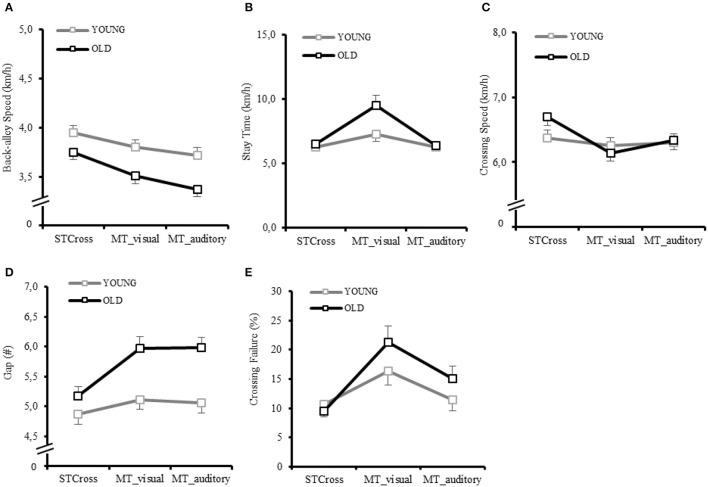
Condition differences between single-task crossing (STCross), multitask typing visually (MTtype_vis) and multitask typing auditorily (MTtype_aud), grouped by age (M and SE) for **(A)** Back-alley Speed; **(B)** Stay Time; **(C)** Crossing Speed; **(D)** Gap Number; **(E)** Crossing Failures.

Table 1 and Figure 2 show descriptive data on each street crossing parameter and Table 2 pertinent ANOVA results.

**Table 1:** Means (M) and Standard Deviations (SD) of crossing parameters during single-task crossing (STCross), multitask typing visually (MTtype_vis) and multitask typing auditorily (MTtype_aud).

	**STCross M (*SD*)**		**MTtype_vis M (*SD*)**		**MTtype_aud M (*SD*)**	
	**YA**	**OA**	**YA**	**OA**	**YA**	**OA**
Stay Time	6.29 (0.97)	6.48 (0.99)	7.26 (4.19)	9.51 (5.99)	6.27 (0.91)	6.41 (1.01)
Back-alley Speed (km/h)	3.94 (0.61)	3.75 (0.59)	3.80 (0.60)	3.51 (0.61)	3.72 (0.61)	3.37 (0.62)
Crossing Speed (km/h)	6.37 (0.97)	6.70 (1.03)	6.26 (0.94)	6.14 (1.02)	6.30 (1.02)	6.33 (1.08)
Crossing Failure (%)	10.63 (16.74)	9.51 (14.19)	16.35 (19.12)	21.31 (21.64)	11.43 (14.69)	15.08 (16.29)
Gap (#)	4.87 (1.32)	5.17 (1.30)	5.11 (1.24)	5.98 (1.53)	5.10 (1.33)	5.98 (1.39)

**Table 2:** ANOVA results for street crossing parameters.

	**df (Error)**	***F***	**Sig**	**$\eta_{p}^{2}$**
**BACK-ALLEY SPEED**				
Condition	1.672 (203.929)	95.064	<0.001^**^	0.438
Age	1 (122)	7.038	0.009^**^	0.055
Condition × Age	1.672 (203.929)	5.930	0.005^**^	0.046
**STAY TIME**				
Condition	1.009 (123.112)	18.344	<0.001^**^	0.131
Age	1 (122)	6.937	0.010^*^	0.054
Condition × Age	1.01 (123.112)	4.855	0.029^*^	0.038
**CROSSING SPEED**				
Condition	2 (244)	40.003	<0.001^**^	0.247
Age	1 (122)	0.220	0.640	0.002
Condition × Age	2 (244)	17.677	<0.001^**^	0.247
**CROSSING FAILURE**				
Condition	1.895 (231.246)	18.423	<0.001^**^	0.131
Age	1 (122)	0.915	0.341	0.007
Condition × Age	1.895 (231.246)	2.407	0.095	0.019
**GAP**				
Condition	2 (244)	33.970	<0.001^**^	0.218
Age	1 (122)	9.228	0.003^**^	0.070
Condition × Age	2 (244)	11.609	<0.001^**^	0.087

* p < 0.05;

** *p < 0.01*.

*Back-alley Speed* was significantly slower in OA than YA and differed between conditions (Figure 2A). It was significantly slower in the MTtype conditions compared to the STCross condition but also differed significantly between both MTtype conditions (slowest in MTtype_aud followed by MTtype_vis and fastest in STCross), especially in OA (significant age × condition interaction). Pairwise *post-hoc* comparisons revealed that age differences only emerged in the MTtype conditions (MTtype_vis: *p* = 0.007; MTtype_aud: *p* = 0.002), but not in STCross *(p* = 0.066).

*Stay time* was significantly longer in OA than YA and different between conditions (Figure 2B). It was significant longer in MTtype_vis compared to MTtype_aud and compared to STCross (always, *p* < 0.001), particularly so in older persons as shown by a significant age by condition interaction. However, *post-hoc* tests revealed significant age differences in MTtype_vis only (*p* = 0.016).

*Crossing Speed* did not differ as a function of age, but again differed between conditions (Figure 2C). Also the age by condition interaction was significant, indicating that only for older adults *Crossing Speed* was significantly affected by condition. OA were significantly slower in both MTtype conditions compared to STcross, but this time with the slowest *Crossing Speed* in MTtype_vis that was also significantly slower compared to the MTtype_aud condition (*p* = 0.001).

*Crossing Failure* revealed a significant condition effect only (Figure 2D)*. Post-hoc* comparisons revealed that this effect was driven by MTtype_vis for which *Crossing Failure* was significantly higher compared to STCross (*p* < 0.001) as well as to MTtype_aud (*p* < 0.001).

OA selected later *Gaps* than YA (significant effect of Age), and gap selection was significant earlier within STcross compared to both MTtype conditions (significant condition effect; both *p* < 0.001) (Figure 2E). As indicated by the age by condition interaction, this condition effect was only driven by OA. Pairwise *post-hoc* comparisons revealed significant age differences in MTtype_vis (*p* = 0.001) and MTtype_aud (*p* < 0.001).

### Typing task

**Figure 3:**
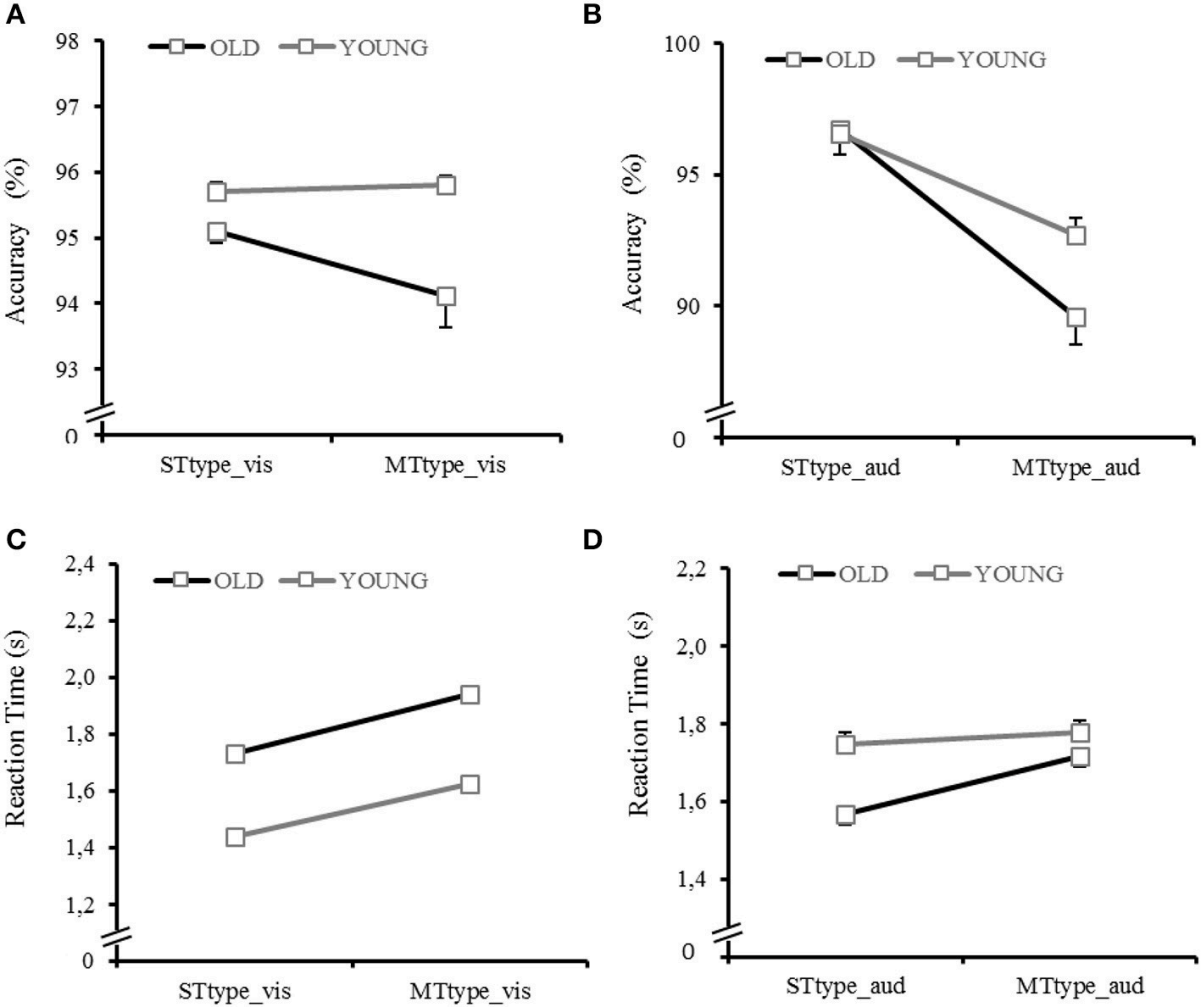
Condition differences between single-task typing visual (STtype_vis) and multitask typing visual (MTtype_vis) and between single task typing auditory (STtype_aud) and multitask typing auditory (MTtype_aud) for **(A)** accuracy visual; **(B)** accuracy auditory; **(C)** reaction time visual; **(D)** reaction time auditory.

Table 3 and Figure 3 show descriptive data on all typing task parameters and Table 4 pertinent ANOVA results.

**Table 3:** Means (M) and Standard Deviations (SD) of typing parameters for younger (YA) and older adults (OA).

	**STTyping_visual M (*SD*)**		**MT_visual M (*SD*)**		**STTyping_auditory M (*SD*)**		**MT_auditory M (*SD*)**	
	**YA**	**OA**	**YA**	**OA**	**YA**	**OA**	**YA**	**OA**
Accuracy in %	95.70 (1.17)	95.10 (1.36)	95.80 (1.13)	94.11 (3,66)	96.58 (3.51)	96.74 (7.80)	92.73 (5.05)	89.61 (8.31)
Reaction Time (s)	1.44 (0.19)	1.73 (0.18)	1.62 (0.24)	1.94 (0.22)	1.75 (0.25)	1.57 (0.21)	1.78 (0.23)	1.72 (0.21)

**Table 4:** ANOVA results for typing parameters.

	***df***	***F***	**Sig**	**$\eta_{p}^{2}$**
**Accuracy**				
Age	1 (122)	8.894	0.004^**^	0.067
Condition	1 (122)	51.217	<0.001^**^	0.296
Condition × Age	1 (122)	6.937	<0.001^**^	0.054
Task Modality	1 (122)	7.992	0.005^**^	0.061
Task Modality × Age	1 (122)	0.143	0.706	0.001
Condition × Task Modality	1 (122)	36.702	<0.001^**^	0.231
Condition × Age × Task Modality	1 (122)	1.728	0.191	0.014
**REACTION TIME**				
Age	1 (122)	9.401	0.003^**^	0.072
Condition	1 (122)	112.852	<0.001^**^	0.481
Condition × Age	1 (122)	6.912	0.010^*^	0.054
Task Modality	1 (122)	1.094	0.298	0.009
Task Modality × Age	1 (122)	145.077	<0.001^**^	0.543
Condition × Task Modality	1 (122)	31.572	<0.001^**^	0.206
Condition × Age × Task Modality	1 (122)	6.283	0.014^*^	0.049

* p < 0.05;

** *p < 0.01*.

*Accuracy* scores for typing were significantly lower in OA than to YA, significantly lower in MTtype conditions than to STtype conditions and significantly lower in the auditory than the visual task modality (Figures 3A,B). The condition by age interaction and corresponding *post-hoc* tests revealed, that age differences only occurred in the MT conditions (*p* < 0.001). Differences between STtype and MTtype conditions occurred for the auditory task modality (*p* < 0.001), while task modality differences occurred within both conditions (significant condition × task modality interaction).

*Reaction Time* was significantly longer for OA than YA (age effect) and longer in MTtype conditions than STtype conditions (condition effect) (Figures 3C,D). This was particularly true for OA as shown by the age × condition interaction. Pairwise comparisons revealed, that age differences only occurred in the MTtype conditions (*p* < 0.001) but within both task modalities (auditory: *p* = 0.001; visual *p* < 0.001; significant age × task modality interaction).

Condition differences were found within both task modalities but task modality differences were only present in STtype condition (significant condition × task modality interaction). Pairwise comparisons for the condition × modality × age interaction revealed that within the visual task modality, age differences were present in the single task (*p* < 0.001) as well as the multitasks (always *p* < 0.001). Within the auditory task modality, age differences were only present in the STtype condition (*p* < 0.001).

### Multitasking effects

Table 5 shows MTE scores for crossing as well as typing parameters, and their differences from zero. Within the visual task modality, significant non-zero MTE scores emerged for both age groups in all street crossing parameters and in *Reaction Time*. Within the auditory task modality, significant non-zero MTE scores in both age groups were only yielded for *Walking Speed, Gap*, and *Accuracy*, while significant non-zero MTE for *Crossing Speed, Crossing Failure*, and *Reaction Time* were only found in OA. These data did not support a consistent relationship between multitasking deficits and task modality.

**Table 5:** Means (M) and Standard Deviations (SD) of multitasking effects within the visual task modality (MTE visual) and within the auditory task modality (MTE auditory), and their difference from zero.

	**MTE visual**		**MTE auditory**	
	**YA**	**OA**	**YA**	**OA**
	**M (*SD*)**	**M (*SD*)**	**M (*SD*)**	**M (*SD*)**
**CROSSING PARAMETERS**				
Stay Time (s)	−25.91 (68.91)^**^	−86.94 (191.59)^**^	−0.79 (6.15)	−1.01 (7.04)
Back-alley Speed (km/h)	−3.56 (5.29)^**^	−6.28 (8.45)^**^	−5.69 (5.08)^**^	−10.05 (8.13)^**^
Crossing Speed (km/h)	−1.61 (4.94)^*^	−8.10 (8.36)^**^	−0.95 (5.66)	−5.48 (6.57)^**^
Crossing Failure (%)	−5.71 (16.24)^**^	−11.80 (19.79)^**^	−7.94 (14.29)	−5.57 (16.38)^*^
Gap (#)	−6.93 (15.65)^**^	−17.59 (24.93)^**^	−5.12 (15.39)^*^	−19.16 (26.91)^**^
**TYPING PARAMETERS**				
Accuracy	0.12 (1.79)	−1.03 (3.87)	−3.69 (5.28)^**^	−7.41 (6.36)^**^
Reaction time	−13.18 (11.52)^**^	−12.74 (13.11)^**^	−2.60 (11.35)	−10.44 (12.19)^**^

* p < 0.05;

** *p < 0.01*.

### Prioritization: street crossing-related MTE vs. typing-related MTE

The ANOVA results for MTE are depicted in Table 6, and the pertinent *post-hoc* comparisons are summarized in Table 7. MTE differed significantly between street crossing-related and typing-related parameters. OA were more likely to produce significant differences between street crossing- and typing-related MTE compared to YA. Further, in the visual task modality significantly higher MTE occurred more frequently for the street crossing-related parameters than for the typing-related ones, especially in OA. This was contrary to findings in the auditory task modality, where significantly higher MTE were produced more frequently in the typing task. However, the direction of those differences was not consistent overall, for a given age group or for a given modality.

**Table 6:** ANOVA results for multitasking effects.

	***df* (Error)**	***F***	**Sig**	**$\eta_{p}^{2}$**
Age	1 (122)	9.566	0.002^**^	0.073
Parameter Type	6 (154.025)	19.637	<0.001^**^	0.139
Parameter Type × Age	6 (154.025)	5.296	0.016^*^	0.042
Task Modality	1 (122)	24.151	<0.001^**^	0.165
Task Modality × Age	1 (122)	0.068	0.068	0.027
Parameter Type × Task Modality	6 (132.331)	18.744	<0.001^**^	0.133
Parameter Type × Age × Task Modality	6 (132.331)	6.180	0.012^*^	0.048

* p < 0.05;

** *p < 0.01*.

**Table 7:** *Post-hoc* comparisons between street crossing-related (rows) and typing-related (columns) multitasking effects, separately for each age group (YA; OA) and task modality.

**MTE (%)**	**Visual task modality**				**Auditory task modality**			
	**YA**	**OA**	**YA**	**OA**	**YA**	**OA**	**YA**	**OA**
	**Reaction Time**	**Reaction Time**	**Accuracy**	**Accuracy**	**Reaction Time**	**Reaction Time**	**Accuracy**	**Accuracy**
Back-alley Speed	<0.001^**^	<0.001^**^	0.003^**^	<0.001^**^	<0.001^**^	<0.001^**^	<0.001^**^	<0.001^**^
Stay Time		0.002^**^		<0.001^**^		0.041^*^	<0.001^**^	<0.001^**^
Crossing Speed	<0.001^**^	<0.001^**^		<0.001^**^		0.001^**^	<0.001^**^	<0.001^**^
Crossing Failure				<0.001^**^				
Gap				<0.001^**^				0.002^**^

* p < 0.05;

** *p < 0.01. Bold, Street crossing MTE > Typing MTE; Italic, Typing MTE > Street crossing MTE*.

**Figure 4:**
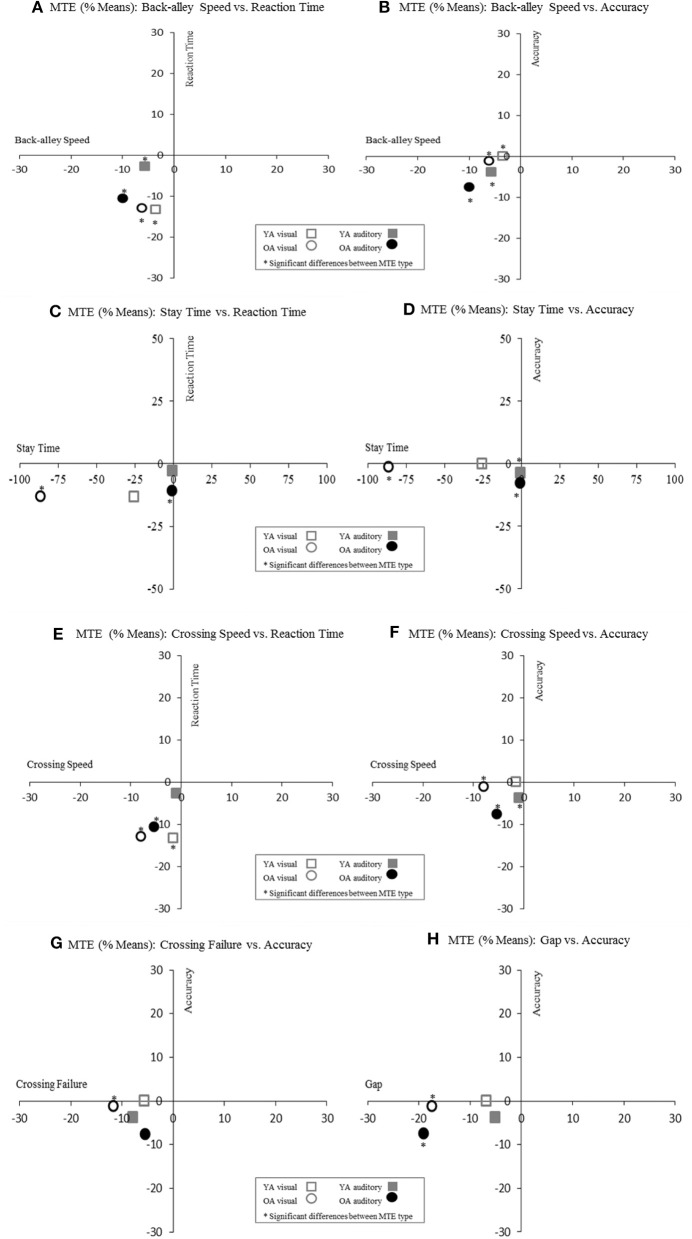
Multitasking Effects (MTE) distribution (Means in %) grouped by age (YA vs. OA) and task type (street crossing related vs. typing related).

These data argued against an overall or an age-dependent prioritization of the street-crossing or the typing task. To emphasize this lack of an overall prioritization strategy, we plotted the Means of significant street crossing-related vs. typing-related MTE differences, grouped by age (see Figure 4).

## Discussion

The aim of this study was to expand available dual-task research by using an ecologically valid task, street crossing in virtual reality, and by including realistic loading tasks and stimulus modalities. The loading task was delivered via two different task modalities (visual vs. auditory) to further provide a theoretical contribution toward the multiple resource theory and Mulittasking Effects were considered to identify possible general prioritization strategies.

We expected to confirm that even in our ecologically valid scenario the costs of multitasking increase in older age and that this increase is more pronounced in the loading tasks as compared to the street crossing task, in accordance with the posture-first hypothesis (Lindenberger et al., 2000; Li et al., 2001; Schäfer et al., 2006). Further we expected that this increase is more pronounced in the visual than the auditory task modality, in accordance with the multiple-resource model (Wickens, 2002).

In accordance with our first expectation, we found that street crossing as well as typing performance suffered under multitasking conditions, and that impairments were more pronounced in older adults. When multitasking, older adults slowed down more than young ones when approaching the curb and when crossing the street, waited longer at the curb, and therefore selected a later gap for crossing. This is in line with previous street crossing studies which also found longer approach durations (Banducci et al., 2016), longer preparation durations (Neider et al., 2010, 2011; Chaddock et al., 2011, 2012; Byington and Schwebel, 2013; Gaspar et al., 2014; Banducci et al., 2016) and more missed crossing opportunities (Stavrinos et al., 2011; Byington and Schwebel, 2013). Our findings are also in line with traditional laboratory studies, which found stronger effects of multitasking on gait speed for older than young persons (Lindenberger et al., 2000; Hausdorff et al., 2008).

The age-related decrement of multitasking abilities manifested not only in four of our five street-crossing parameters, but also in both loading-task parameters. This conforms earlier findings about differential effects of age on loading-task performance (Lindenberger et al., 2000; Li et al., 2012), and extends them to an ecologically valid scenario.

Surprisingly, we did not find age-related decrements in *Crossing Speed*, even though a reduced walking speed in OA compared to YA under dual-task conditions has been reported in dual-task gait studies before (Lindenberger et al., 2000; Hausdorff et al., 2008). In contrast, Neider et al. (2011) reported even smaller crossing durations (i.e., faster crossing speeds) in OA as compared to YA and interpreted this finding as a greater perceived urge in OA to avoid (virtual) collisions. In our study, both age groups crossed the street very quickly (around 6 km/h) which means that especially OA accelerated their regular walking speed (4.6–4.9 km/h; Samson et al., 2001) and invested more motor (and cognitive) resources in order to complete the crossing as fast as possible. Especially in a demanding multitasking situation, this additional invest of resources might exceed the limits of their processing capacity.

In accordance with our second expectation, multitasking had more pronounced effects on the loading tasks than on the street-crossing task, but this was the case for only about half of the age × modality × parameter combinations (cf. Table 5). For the other half, multitasking had more pronounced effects on the street-crossing task. This heterogeneity persists even when only the two crossing parameters with the closest link to posture and gait are considered, namely, *Back-alley Speed* and *Crossing Speed*. From this we conclude that the posture-first hypotheis may not be applicable unconditionally (Li et al., 2012), and in ecologically valid scenarios. These heterogeneous results could either indicate implicit, individual prioritization strategies or a limitation due to task difficulties. Thus, participants may either have not perceived a risk to their health out of the virtual reality which would limit the extent to what VR scenarios transmit a real life impression or might have been limited by ceiling effects within some tasks. Overall, it appears that realistic loading tasks can be motivating enough to override older persons' concerns about postural stability. However, it has to be mentioned that in our study, participants were allowed to keep one hand to the treadmill's handrail which might have influenced participant's perceived postural control. In this vein, Lövdén et al. (2005) revealed that older adults' navigation performance improves when holding on to a handrail. Thus, future studies should systematically investigate the influence of additionl support and might also assess general as well as test set up-related anxiety scores such as fear of falls.

Inconsistent with our third expectation, effects of multitasking were not consistently more pronounced in the visual compared to the auditory modality (cf. Tables 3, 4). More pronounced multitasking effects in the visual than auditory condition are in accordance with an earlier virtual-driving study where visual and auditory loading tasks were presented blockwise (Chaparro et al., 2005). In our study, however, stronger effects of the visual modality were observed for only a part of the age × parameter combinations. This was most striking for *Stay Time*, for which the visual task modality (in YA as well as in OA) caused the highest overall MTE of all parameters, implicating that within this phase of the crossing process, vision might play an indispensable role. However, for other combinations, both modalities yielded similar effects or the auditory modality even yielded stronger effects e.g., for *Back Alley Speed* which was surprisingly more effected by the auditory task modality. We therefore found no unequivocal support for the multiple resource model in our ecologically valid scenario. Possibly, our multitasking scenario was complex enough to give participants a choice exactly what resources they allocated to the task at hand. As a consequence, participants' strategic choices could have upset any strict relationship between task modality and multitasking effects.

The lack of a consistent relationship between task modality and multitasking performance is particularly striking for our loading task: multitasking effects on *Typing Reaction Time* were more pronounced in the visual modality, while those on *Typing Accuracy* were stronger in the auditory modality. We contribute this particular dissimilarity to the fact that in the German language, the ten's and one's of numbers are spoken in reversed order (e.g., “two hundred and five-and-forty” instead of “two hundred and forty-five”). If participants pressed the keys in the same order in which digits were spoken, this would have reduced.

In conclusion, our findings confirm that age-related deficits of multitasking exist even in ecologically valid scenarios, and document that those deficits can emerge in both concurrent tasks. However, our findings provide no unequivocal support for the posture-first hypothesis and for the multiple-resource model. We attribute this lack of support to motivational and to strategic factors, which are controlled for in traditional laboratory paradigms but play a major role in realistic behavior.

## Author contributions

CJ performed the acquisition of data and data analyses and wrote the drafts of the manuscript. CV-R as a senior author as well as OB contributed in terms of data selection, data analysis as well as in editing the manuscript and providing additional ideas at how to interpret our data. KW, UD and MH supported the data collection as well as the data analysis and proof read the manuscript.

### Conflict of interest statement

The authors declare that the research was conducted in the absence of any commercial or financial relationships that could be construed as a potential conflict of interest.
